# Use of Social Media by Hospitals and Clinics in Japan: Descriptive Study

**DOI:** 10.2196/18666

**Published:** 2020-11-27

**Authors:** Yuya Sugawara, Masayasu Murakami, Hiroto Narimatsu

**Affiliations:** 1 Institute for Promotion of Medical Science Research Faculty of Medicine Yamagata University Yamagata Japan; 2 Department of Health Policy Science Graduate School of Medical Science Yamagata University Yamagata Japan; 3 Cancer Prevention and Control Division Kanagawa Cancer Center Research Institute Yokohama Japan; 4 Graduate School of Health Innovation Kanagawa University of Human Services Kawasaki Japan

**Keywords:** social media, internet, hospitals, health promotion, Japan

## Abstract

**Background:**

The use of social media by hospitals has become widespread in the United States and Western European countries. However, in Japan, the extent to which hospitals and clinics use social media is unknown. Furthermore, recent revisions to the Medical Care Act may subject social media content to regulation.

**Objective:**

The purpose of this study was to examine social media use in Japanese hospitals and clinics. We investigated the adoption of social media, analyzed social media content, and compared content with medical advertising guidelines.

**Methods:**

We randomly sampled 300 hospitals and 300 clinics from a list of medical institutions that was compiled by the Ministry of Health, Labour and Welfare. We performed web and social media (Facebook and Twitter) searches using the hospital and clinic names to determine whether they had social media accounts. We collected Facebook posts and Twitter tweets and categorized them based on their content (eg, health promotion, participation in academic meetings and publications, public relations or news announcements, and recruitment). We compared the collected content with medical advertising guidelines.

**Results:**

We found that 26.0% (78/300) of the hospitals and 7.7% (23/300) of the clinics used Facebook, Twitter, or both. Public relations or news announcements accounted for 53.99% (724/1341) of the Facebook posts by hospitals and 58.4% (122/209) of the Facebook posts by clinics. In hospitals, 16/1341 (1.19%) Facebook posts and 6/574 (1.0%) tweets and in clinics, 8/209 (3.8%) Facebook posts and 15/330 (4.5%) tweets could conflict medical advertising guidelines.

**Conclusions:**

Fewer hospitals and clinics in Japan use social media as compared to other countries. Social media were mainly used for public relations. Some content disseminated by medical institutions could conflict with medical advertising guidelines. This study may serve as a reference for medical institutions to guide social media usage and may help improve medical website advertising in Japan.

## Introduction

More than 4.5 billion people use the internet, and the number of social media users worldwide has passed the 3.8 billion mark as of the start of 2020 [1]. Facebook and Twitter are popular social media tools. As of the second quarter of 2020, Facebook had over 2.7 billion monthly active users (MAUs) [2]. As of the first quarter of 2019, Twitter had an average of 330 million MAUs worldwide [3]. In Japan, in 2019, Facebook had 26 million MAUs, and Twitter had about 48 million MAUs [4].

A major benefit of social media for health communication is the accessibility and widening access of health information to various population groups, regardless of age, education, race or ethnicity, and locality [5]. Thus, many health care organizations use social media. In the United States, 94.41% of hospitals have Facebook pages, and 50.82% have Twitter accounts [6]. In Western Europe, 67.0% of hospitals have Facebook pages, and 18.1% have Twitter accounts [7]. Thaker et al [8] reported that hospitals use social media to announce news and events and to promote themselves and health.

While many hospitals disseminate beneficial health information, there is concern that some hospital social media content may breach patient privacy [9]. Some hospitals may disseminate blatant advertising [10]. Some plastic surgeons emphasize immediate positive results, without discussing any potential complications or postoperative care requirements, and photoshopped before and after pictures are commonplace in some social media posts [10]. Japan’s Medical Care Act was amended in 2017 and the new Medical Care Act has been enforced since 2018 [11,12]. With this revision, websites of medical institutions that were previously not considered advertisements are now considered so and are also now subject to regulation. To this end, administrative and criminal penalties have been introduced for violations. In addition, medical advertisement guidelines were also revised by the Ministry of Health, Labour and Welfare (MHLW) [13]. The contents of health care organizations’ websites have been restricted because of these revisions. Although the guidelines do not mention social media, they may be identified as websites, and the contents of health care organizations’ social media can thus be restricted. However, Japanese hospitals and clinics may disseminate health information that do not follow medical advertisement guidelines.

Presently, the extent to which Japanese health care organizations use social media is unknown, necessitating investigation. Accordingly, this study was designed to investigate the outline of social media use in Japanese hospitals and clinics through the following research questions:

1. How many social media accounts do Japanese medical institutions have?

2. What kind of information do Japanese medical institutions post on social media?

3. Does the information posted by the medical institutions conform to the medical advertising guidelines?

## Methods

### Study Population

We extracted study samples based on lists that were available. The list of insurance-covered medical institutions is maintained by the Regional Bureau of Health and Welfare, MHLW [14,15]. The list of clinics that performed treatment not covered by health insurance was published on Yahoo! Healthcare [16]. We extracted 8600 hospitals, 154,213 clinics, and 515 clinics that performed treatment not covered by health insurance, from the lists of medical institutions.

In Japan, the universal insurance system was established in 1961 [17]. This system allows anyone to visit medical institutions anytime and anywhere with no discrimination [18,19]. Therefore, we assumed that there was no difference in regional medical care provision and the use of social media. In this study, 300 samples were uniformly extracted from hospitals and clinics in Japan without considering regional bias. We assigned a random number to each hospital and clinic using an Excel (Microsoft) function. After assigning a random number, 300 samples were extracted in the descending order of random numbers. The size of the extracted sample was estimated based on the interval estimation of the population proportion. We performed a pilot study from February 23, 2018, to March 12, 2018, extracting 200 samples for trial. The results indicated that 26.5% (53/200) of hospitals used social media. We estimated that the sample size was 300 by using the statistical software EZR with the width of the 95% confidence interval as 0.1, so that the actual results fit within ±5% of the true value with the expected proportion being 0.265. Moreover, the 200 test samples were not included in the 300 samples used in the main study.

The date of designation as insurance-covered medical institutions, the name of the medical institutions, address, phone number, ID of medical institutions, and specialty are contained in the list of insurance-covered medical institutions. This list has been published on a website maintained the Regional Bureau Health and Welfare of each region [20-27]. Anyone can freely download the list as a PDF file (Adobe) or MS Excel file. We used the data of insurance-covered medical institutions as of October 1, 2017, in this study. We accessed Yahoo! Healthcare to collect the data not covered by health insurance clinics on November 5, 2017. However, Yahoo! Healthcare, which published information on health care and medical institutions on its website, was shut down on March 29, 2018 [16].

### Social Media Accounts of Hospitals and Clinics

Facebook and Twitter, the major social media in Japan, were selected for analysis. For each sample of 300 hospitals and clinics, we performed Google searches using the names of the hospitals and clinics. We checked whether social media accounts of hospitals and clinics exist. Using the search engine on the official social media page as well as Google, the name of each medical institution was searched to check for the existence of a social media account. For medical institutions that have social media accounts, their websites were checked to see whether a social media policy has been formulated.

We surveyed the numbers of “likes” and “followers” from the medical institutions’ Facebook and Twitter pages. The attributes of each hospital and clinic (clinical department, number of beds, types of beds, who established it) were drawn from the extracted hospitals and clinics’ websites and the list of insurance-covered medical institutions.

The survey of the social media accounts of hospitals and clinics was conducted from April 7 to April 22, 2018. We accessed social media accounts of hospitals from April 7 to 15, 2018, and clinics from April 15 to 19, 2018. The data gathering of social media accounts was completed on April 22, 2018.

### Data Collection

We collected content from Facebook and Twitter. For each hospital and clinic account, we collected 100 Facebook posts and 1000 tweets. Content data were collected using NodeXL Excel Template 2014 (version 1.0.1.402; The Social Media Research Foundation), an MS Excel add-in [28]. After collecting Facebook posts and tweets, to investigate the difference in the number of comments for each season, the number of monthly comments in 2017 for each hospital and clinic was calculated. Then, Facebook post and Twitter tweet data were collected between August 4 and 5, 2018.

### Classification of Contents

The latest 20 Facebook posts and the latest 100 tweets were manually categorized by content per medical institution. Contents were categorized manually and classified into 4 types: “Health promotion,” “Participation in academic meetings, publications,” “Public relations, news announcements,” and “Recruitment.” At first, it was divided into “Health promotion,” “Public relations, news announcements,” and “Recruitment” with reference to previous studies [5,8,29,30]. As we continued the classification, we found that there was a lot of content related to participation in academic meetings and publications. Therefore, a new item “Participation in academic meetings, publications” was added. We categorized social media contents as shown in Textbox 1.

If the social media content was updates on the medical institution’s blog, we checked the links and categorized the comments. If more than 1 content is included, the main topic is judged from the context and the comments are categorized.

Three researchers (a medical informatics specialist [YS], a health policy specialist [MM], a medical doctor and public health specialist [HN]) categorized contents into 4 types. When a conflict occurred, it was resolved by discussions between the 3 researchers. Thus, all content was categorized upon agreement from the 3 researchers.

**Social media contents.**

- Health Promotion: Dissemination of medical knowledge and health information. This includes easy-to-understand medical knowledge and health information for patients and the public, and professional information for professionals.
- Participation in Academic Meetings, Publications: Comments on academic activities such as information on holding academic meetings, participation in academic meetings, writing papers, and specialized books.
- Public Relations, News Announcements: Reports on in-hospital events for patients, notifications from hospitals, public relations, comments related to consultation (eg, hospitals are closed, change in consultation hours).
- Recruitment: Content related to human resources, such as personnel change reports and comments on recruitment.
- Others: Comments that do not apply to any of the above. For example, comments on activities that are not related to the actual work, such as welcome parties, social gatherings, and sports competitions.

### Comparison With Guidelines

We compared the collected contents with the medical advertising guidelines and examined whether they complied with the guidelines or were appropriate as advertisements. In addition to the medical advertising guidelines, the “Doctor’s Professional Ethics Guidelines,” “The way medical facility websites should be – Guidelines for providing member medical facilities and medical information” (2008 March revised edition; both issued by the Japan Medical Association), and a previous study that compared medical advertising guidelines and the websites of medical institutions related to aesthetic medicine were used to create evaluation items and criteria (Multimedia Appendix 1) [31-33]. Referring to the criteria and the advertising example described in the medical advertising guidelines, 3 researchers (a medical informatics specialist [YS], a health policy specialist [MM], a medical doctor and public health specialist [HN]) compared contents and medical advertising guidelines. Based on the agreement of the 3 researchers, it was decided whether it was appropriate as a medical advertisement.

### Text Mining

To complement manual content analysis, text mining was performed on Facebook posts and tweets of the hospitals and clinics, respectively. We calculated term frequency—which is the number of occurrences of each target word in an entire text—and created a co-occurrence network. We used KH Coder Version 3.Beta.01g for Windows for this task [34-36]. ChaSen, which was used for the morphological analysis, was included in KH Coder and used for word extraction. KH Coder uses the Jaccard coefficient to determine the degree of word-to-word co-occurrence and creates a network chart [37]. In this chart, words closely associated with each other are connected with lines [37]. KH Coder also displays networks that are more closely associated with each other as “subgraphs” through color coding [37]. In this context, co-occurrence means there is a close relationship between words [38].

### Statistical Analysis

The percentage of the social media account holding ratio for each medical institution was calculated. We regarded a medical institution that has either or both a Facebook and Twitter account as “Having a social media account.” We calculated the median and IQR for the numbers of beds, Facebook likes, and Twitter followers.

Fisher exact test and logistic regression analysis were performed on the attributes of medical institutions and whether medical institutions have social media accounts.

Hospital attributes were hospital size (small and medium hospitals with 20-199 beds, large hospitals with more than 200 beds), urban/rural, hospital classification (general hospital, internal medicine hospital, surgical hospital), who established it (individual/nonprofit medical corporations, national/public/social insurance-related organizations), Regional Bureau of Health and Welfare in each region, hospital functions (general hospitals, special functioning hospitals or regional medical care support hospitals), and whether the hospital has a website.

Clinic attributes were whether the clinic has a bed, urban/rural, medical/dental classification, Regional Bureau of Health and Welfare in each region, who established it (individuals, nonprofit medical corporations, national/public), specialty (internal medicine departments, surgical departments, dentistry), and whether the clinic has a website.

In the logistic regression analysis, the presence or absence of social media accounts was analyzed as a dependent variable, and the attributes of medical institutions were analyzed as independent variables.

We compared the ratio of sample medical institutions by region with actual medical institutions. The goodness-of-fit test was performed by the chi-square test with reference to the reports released by the MHLW [39].

A *P* value <.05 was considered statistically significant. Statistical analyses were performed with EZR (version 1.37, Saitama Medical Center, Jichi Medical University), which is a graphical user interface for R (The R Foundation for Statistical Computing). More precisely, it is a modified version of R commander designed to add statistical functions frequently used in biostatistics [40].

This study was approved by the Institutional Review Board of Yamagata University, Faculty of Medicine.

## Results

### Sample Medical Institutions

We extracted 600 medical institutions (300 hospitals and 300 clinics). Of the 300 hospitals, 209 were small and medium hospitals, and 91 were large hospitals; 10 hospitals were special functioning hospitals or regional medical care support hospitals. Of the 300 clinics, 176 were medical clinics and 124 were dental clinics. For the ratio of number of sample medical institutions to the actual number of medical institutions by each Regional Bureau of Health and Welfare, a chi-square test revealed no significant difference in hospitals (*P*=.268, χ^2^_7_=8.791) or clinics (*P*=.958, χ^2^_7_=2.028). Multimedia Appendix 2 shows a table comparing the ratio of sample medical institutions to actual medical institutions.

### Research Question 1

#### Hospital Accounts

Table 1 shows the number and ownership of social media accounts of medical institutions. Of the 300 hospitals and clinics, 78 (26.0%) and 23 (7.7%), respectively, have Facebook or Twitter accounts or both.

Tables 2 and 3 show the results of Fisher exact test and logistic regression analysis for the use of social media and the attributes of hospitals, respectively. The Fisher exact test showed a significant difference in the presence or absence of social media and hospital size (*P*<.001), hospital classification (*P*=.018), hospital function (*P*=.004), and website presence (*P*=.025). Logistic regression analysis showed a significant difference in hospital size (*P*<.001). The odds ratio was 3.25 with a 95% confidence interval ranging from 1.75 to 6.04. No significant difference was found except for hospital size. The ranges of all generalized variance inflation factor in the logistic regression analysis ranged from 1.00 to 1.39.

**Table 1:** Numbers and percentages of social media accounts and websites that medical institutions had (N=300).

Institutions	Social media	Facebook	Twitter	Website
Hospitals, n (%)	78 (26.0)	73 (24.3)	13 (4.3)	286 (95.3)
Clinics, n (%)	23 (7.7)	19 (6.3)	11 (3.7)	129 (43.0)

**Table 2:** Fisher exact test regarding the use of social media and the attributes of medical institutions (hospitals).

Item and Classification		Not using social media (N=222)	Using social media (N=78)	*P* value
**Hospital size**				**<.001**
	Small and medium hospitals with 20-199 beds, n (%)	171 (77.0)	38 (48.7)	
	Large hospitals with more than 200 beds, n (%)	51 (23.0)	40 (51.3)	
**Urban, rural**				**.364**
	Rural, n (%)	18 (8.1)	9 (11.5)	
	Urban, n (%)	204 (91.9)	69 (88.5)	
**Hospital classification**				**.018**
	General hospital, n (%)	108 (48.6)	51 (65.4)	
	Internal medicine hospital, n (%)	99 (44.6)	21 (26.9)	
	Surgical hospital, n (%)	15 (6.8)	6 (7.7)	
**Established by**				**.073**
	Individual/nonprofit medical corporations, n (%)	182 (82.0)	56 (71.8)	
	National/public/social insurance-related organizations, n (%)	40 (18.0)	22 (28.2)	
**Regional Bureau of Health and Welfare**				**.233**
	Hokkaido, n (%)	22 (9.9)	4 (5.1)	
	Tohoku, n (%)	15 (6.8)	6 (7.7)	
	Kanto-Shinetsu, n (%)	48 (21.6)	28 (35.9)	
	Tokai-Hokuriku, n (%)	26 (11.7)	8 (10.3)	
	Kinki, n (%)	43 (19.4)	10 (12.8)	
	Chugoku-Shikoku, n (%)	21 (9.5)	10 (12.8)	
	Shikoku, n (%)	9 (4.1)	3 (3.8)	
	Kyushu, n (%)	38 (17.1)	9 (11.5)	
**Hospital function**				**.004**
	General hospital, n (%)	219 (98.6)	71 (91.0)	
	Special functioning hospitals or regional medical care support hospitals, n (%)	3 (1.4)	7 (9.0)	
**Website**				**.025**
	Absent, n (%)	14 (6.3)	0 (0.0)	
	Present, n (%)	208 (93.7)	78 (100.0)	
Beds, median (IQR)		120.00 (69.25-198.75)	220.00 (100.50-370.00)	<.001
Facebook likes, median (IQR)		N/A^a^	66.00 (19.00-207.00)	
Twitter followers, median (IQR)		N/A	7.00 (3.00-84.00)	

^a^NA: not applicable.

**Table 3:** Logistic regression analysis of hospital attributes and social media usage (hospitals).

Variable		Odds ratio (95% confidence interval)	*P* value
**Hospital size**			
	Small and medium hospitals with 20 to 199 beds	Reference	<.001
	Large hospitals with more than 200 beds	3.25 (1.75-6.04)	
**Hospital classification**			
	General hospital	Reference	
	Internal medicine hospital	0.57 (0.30-1.09)	.088
	Surgical hospital	1.49 (0.51-4.32)	.46
**Established by**			
	Individual/nonprofit medical corporations	Reference	
	National/public/social insurance-related organizations	0.73 (0.34-1.56)	.41
**Hospital function**			
	General hospital	Reference	
	Special functioning hospitals or regional medical care support hospitals	3.27 (0.75-14.40)	.12
**Website**			
	Absent	Reference	
	Present	9200000.00 (0.00-infinity)	.99

#### Clinic Accounts

The number of Facebook and Twitter accounts of clinics was 19/300 (6.3%) and 11/300 (3.7%), respectively (Table 1). Tables 4 and 5 show the results of Fisher exact test and logistic regression analysis. The Fisher test showed a significant difference in website (*P*<.001). Logistic regression analysis showed a significant difference in website (*P*<.001) and specialty (dentistry, *P*=.037). Generalized variance inflation factor in logistic regression analysis was 1.01.

**Table 4:** Fisher exact test regarding the use of social media and the attributes of medical institutions (clinics).

Item and classification		Not using social media (N=277)	Using social media (N=23)	*P* value
**Beds**				**.637**
	Absent, n (%)	261 (94.2)	21 (91.3)	
	Present, n (%)	16 (5.8)	2 (8.7)	
**Urban/Rural**				**.615**
	Rural, n (%)	15 (5.4)	0 (0.0)	
	Urban, n (%)	262 (94.6)	23 (100.0)	
**Medical/Dental classification**				**.13**
	Dental clinics, n (%)	111 (40.1)	13 (56.5)	
	Medical clinics, n (%)	166 (59.9)	10 (43.5)	
**Regional Bureau of Health and Welfare**				**.408**
	Hokkaido, n (%)	7 (2.5)	2 (8.7)	
	Tohoku, n (%)	18 (6.5)	0 (0.0)	
	Kanto-Shinetsu, n (%)	110 (39.7)	9 (39.1)	
	Tokai-Hokuriku, n (%)	36 (13.0)	1 (4.3)	
	Kinki, n (%)	46 (16.6)	4 (17.4)	
	Chugoku-Shikoku, n (%)	15 (5.4)	2 (8.7)	
	Shikoku, n (%)	10 (3.6)	1 (4.3)	
	Kyushu, n (%)	35 (12.6)	4 (17.4)	
**Established by**				**.736**
	Individual, n (%)	167 (60.3)	13 (56.5)	
	Nonprofit medical corporations, n (%)	107 (38.6)	10 (43.5)	
	National/public/social insurance related organizations, n (%)	3 (1.1)	0 (0.0)	
**Specialty**				**.185**
	Internal medicine departments, n (%)	95 (34.3)	4 (17.4)	
	Surgical departments, n (%)	71 (25.6)	6 (26.1)	
	Dentistry, n (%)	111 (40.1)	13 (56.5)	
**Website**				**<.001**
	Absent, n (%)	169 (61.0)	2 (8.7)	
	Present, n (%)	108 (39.0)	21 (91.3)	
Beds, median (IQR)		0.00 (0.00-0.00)	0.00 (0.00-0.00)	.57
Facebook likes, median (IQR)		N/A^a^	69.00 (24.50-95.50)	
Twitter follower, median (IQR)		N/A	11.00 (3.00-23.50)	

^a^NA: not applicable.

**Table 5:** Logistic regression analysis of hospital attributes and social media usage (clinics).

Variable		Odds ratio (95% confidence interval)		*P* value
**Website**				
	Absent	Reference		
	Present	17.80 (4.07-78.20)		<.001
**Specialty**				
	Internal medicine departments	Reference		
	Surgical departments	2.20 (0.58-8.40)		.25
	Dentistry	3.55 (1.08-11.70)		.037

#### Social Media Policy

Three hospitals and no clinics disclosed social media usage policies on their website.

### Research Question 2

#### Number of Comments

**Figure 1:**
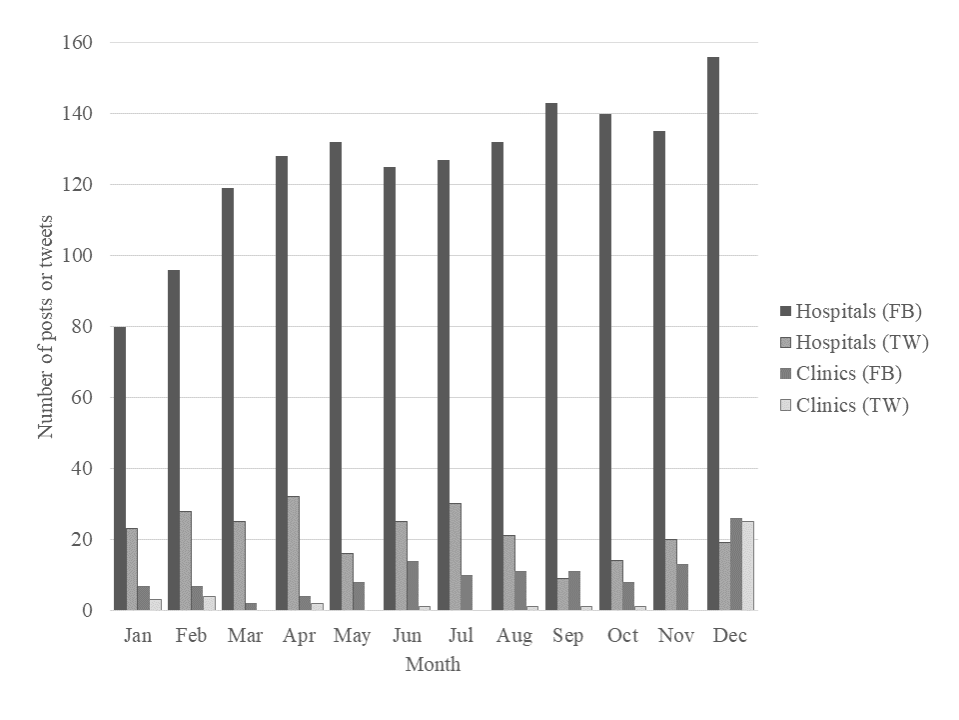
The number of comments for hospitals and clinics in 2017. The number of comments from the clinic was small. The number of comments increased in December at both hospitals and clinics. FB: Facebook; TW: Twitter.

The total number of social media messages disseminated by medical institutions was 8026 from September 16, 2010, to August 4, 2018. Hospitals published 4514 Facebook posts and 2679 tweets, whereas clinics had 503 Facebook posts and 330 tweets. The number of comments we used for content analysis was 1341 hospital Facebook comments and 574 Twitter comments; for clinics, 209 Facebook comments, and 330 Twitter comments. Figure 1 shows the number of monthly comments for the year 2017. For both hospitals and clinics, Facebook posts and tweets all increased in December. In 2017, the annual number of comments for hospitals was 1513 Facebook comments and 262 Twitter comments; for clinics, 121 Facebook comments and 38 Twitter comments. Multimedia Appendix 3 shows examples of contents of hospitals and clinics.

#### Classification of Contents

**Figure 2:**
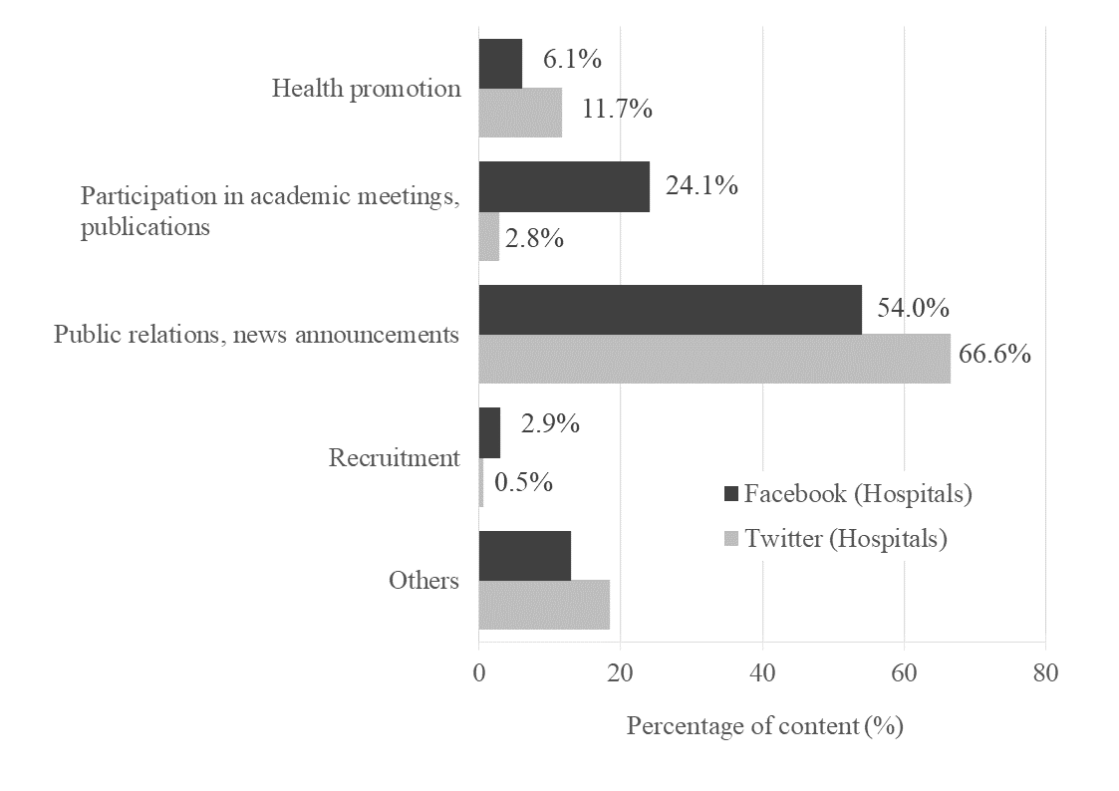
Classification and percentage of social media messages (Hospitals). The latest 20 Facebook posts and the latest 100 tweets were manually categorized by content per medical institution. “Participation in academic meetings, publications” accounted for 24.1% of the Facebook posts.

**Figure 3:**
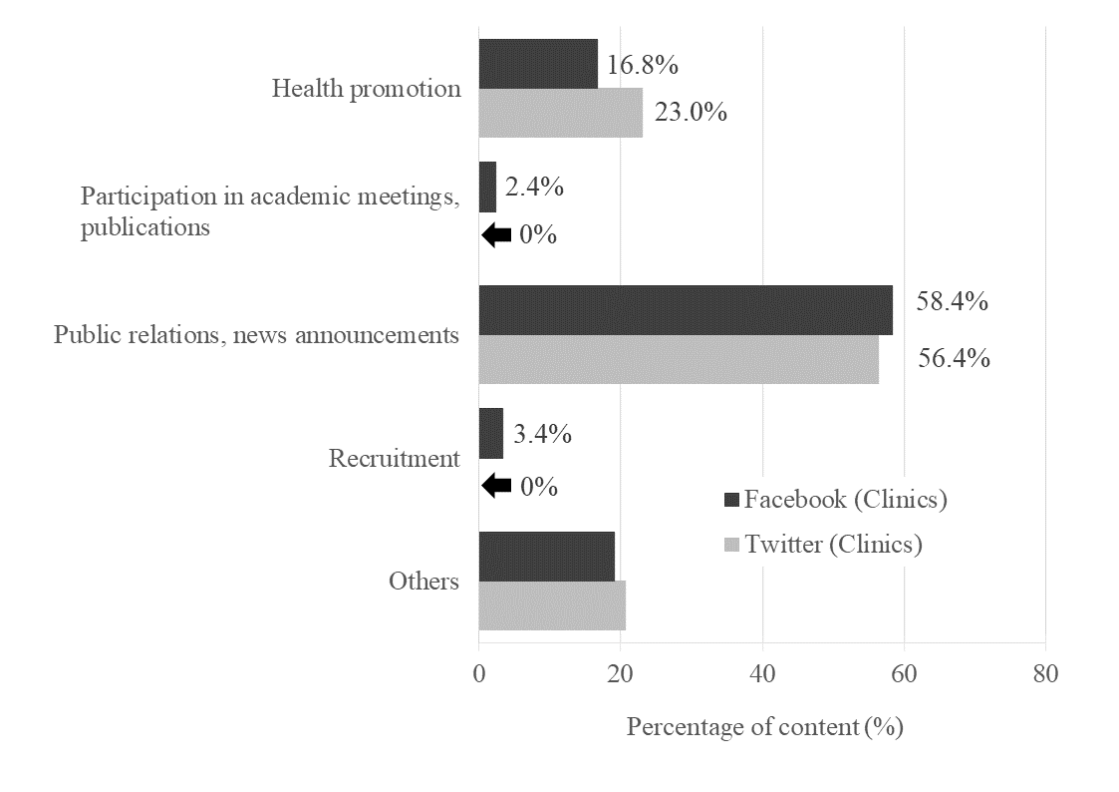
Classification and percentage of social media messages (clinics). The latest 20 Facebook posts and the latest 100 tweets were manually categorized by content per medical institution. Higher percentage of “Health promotion” compared to hospitals.

Figures 2 and 3 show the classification of Facebook and Twitter content of hospitals and clinics, respectively. For hospitals and clinics, “Public relations, news announcement” was the highest, accounting for more than 50% of the content (hospital Facebook content: 53.99% [724/1341]; hospital Twitter content: 66.6% [382/574]; clinic Facebook content: 58.4% [122/209]; clinic Twitter content: 56.4% [186/330]). Compared to hospitals, clinics had posted more “Health promotion” tweets on Twitter. For hospitals using Facebook, “Participation in academic meetings, publications” accounted for 24.09% (323/1341) of the posts, but few in hospitals using Twitter and clinics. Hospitals and clinics disseminated little content related to “Recruitment” on Facebook and Twitter.

#### Term Frequency and Co-occurrence Network

**Figure 4:**
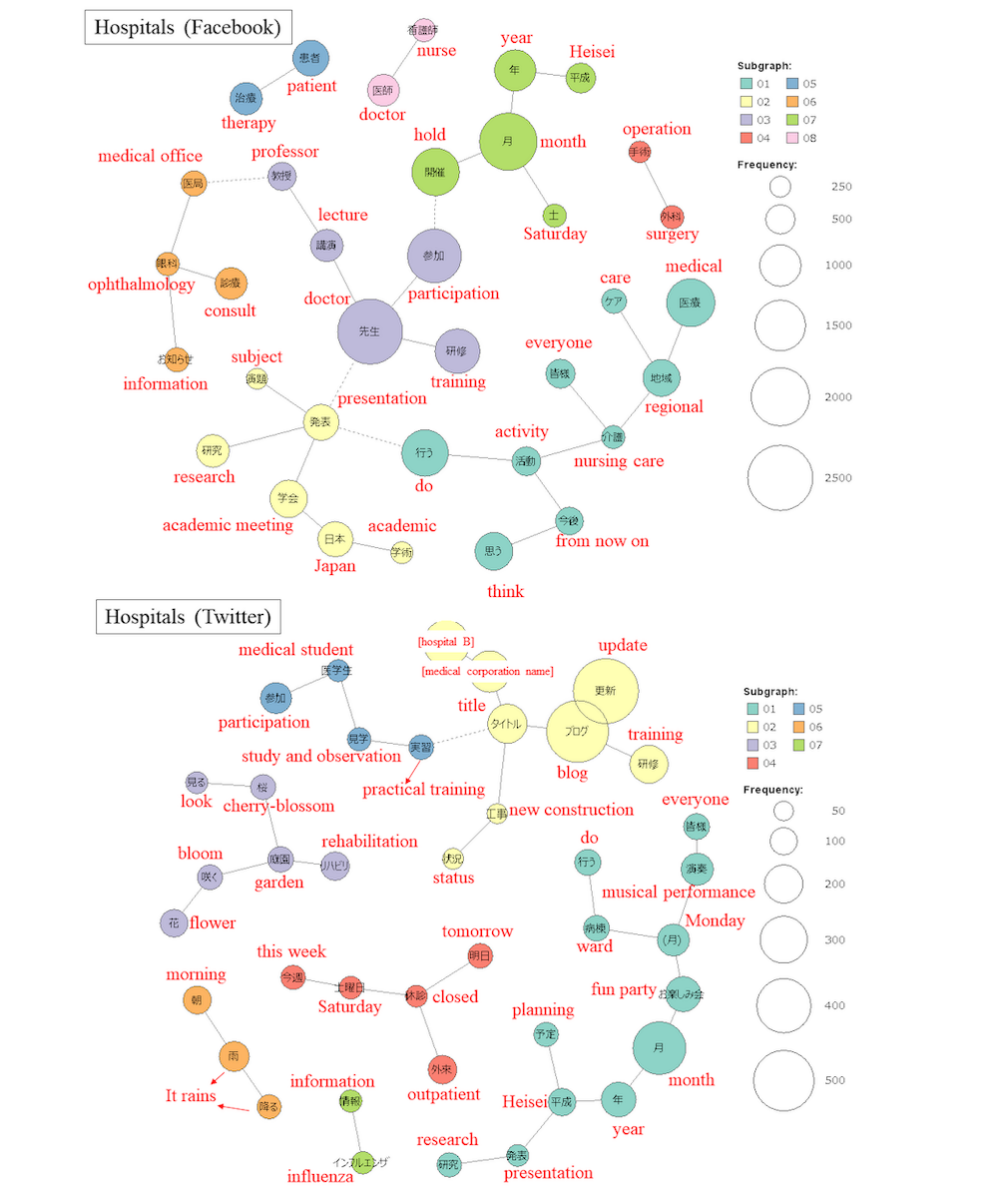
A co-occurrence network for the hospitals in this study. Words in the same subgraph are connected by a solid line. When co-occurring with words in other subgraphs, they are connected by a broken line. Information related to nursing care, community-based health care, academic meeting, and lectures was posted on Facebook. On Twitter, there were tweets about a fun party at a hospital and tweets about updating the blog of hospital B.

**Figure 5:**
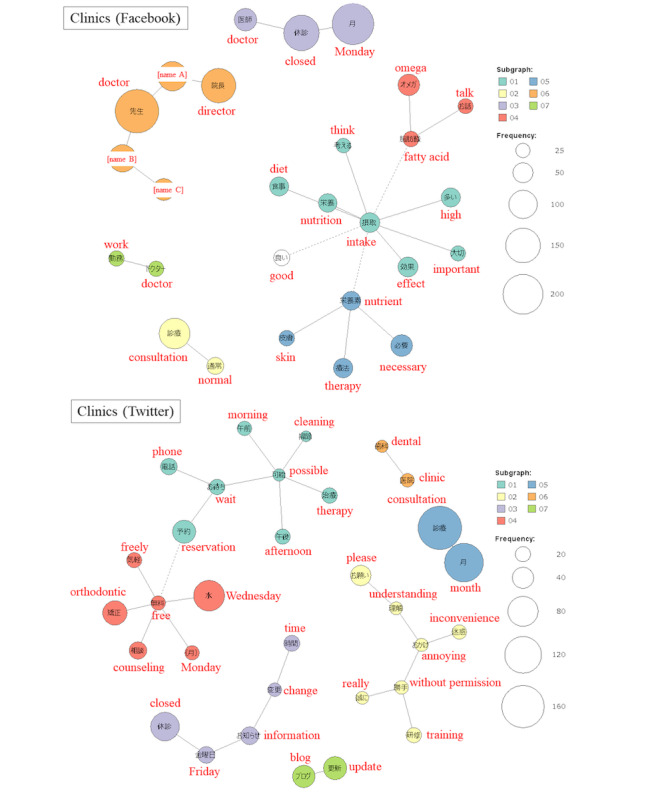
A co-occurrence network for the clinics in this study. Words in the same subgraph are connected by a solid line. When co-occurring with words in other subgraphs, they are connected by a broken line. Single words that do not belong to any subgraph are shown in white. On Facebook, a clinic was raising awareness about nutrition and omega fatty acids. On Twitter, there were tweets about free counseling on orthodontics.

The results of text mining are shown in Figures 4 and 5, and Multimedia Appendix 4. On the Facebook accounts of hospitals, more words related to conference presentations appeared than others. The frequency was 815 times for “academic meeting,” 746 times for “presentation,” and 635 times for “research,” thus, forming a co-occurrence network. On hospital Twitter accounts, “influenza” formed a co-occurrence network. At clinics, there were many announcements about leave of absence on both Facebook and Twitter. The number of occurrences of “closed” was 158 and 73, respectively, on Facebook and Twitter.

### Research Question 3

Table 6 shows the comparison between social media contents and guidelines. Content that could conflict with the guidelines and the percentage of total content by hospitals using Facebook and Twitter were 16 (1.19%, 16/1341) and 6 (1.0%, 6/574), respectively. In clinics, 8 Facebook posts (3.8%, 8/209) and 15 tweets (4.5%, 15/330) could conflict medical advertising guidelines. Multimedia Appendix 5 shows examples of this content.

**Table 6:** Number of messages that may violate medical advertising guidelines and professional ethics.

Evaluation items^a^	Applicable part of the guidelines^b^	Hospitals		Clinics	
		Facebook	Twitter	Facebook	Twitter
Introduction in media	By quoting or publishing articles in newspapers and magazines, discourses, theories, and experiences of doctors and scholars	12	4	4	0
Messages on safety	Misleading advertising	0	0	1	0
Invitation by matters not related to providing medical care	Advertising that impairs dignity	0	0	0	5
Emphasis on cost	Advertising that impairs dignity	0	2	1	10
Medical department name	Not included in advertisable items	0	0	0	0
Professional qualification	Not included in advertisable items	1	0	0	0
Regulations by other laws and regulations	Advertising prohibited by other laws or other advertising guidelines	1	0	0	0
Messages suggesting the superiority of the medical institution by comparison, exaggerated expressions of facility size, staffing, or medical provision	Advertising that implies their superiority by comparison/Misleading advertising	0	0	2	0
Ethical issues	Refer to the “Doctors’ Professional Ethics Guidelines” issued by the Japan Medical Association	2	0	0	0

^a,b^Refer to Multimedia Appendix 1.

## Discussion

### Preliminary Findings

In this study, 300 hospitals and clinics, respectively, were sampled and classified according to social media accounts and their contents. In Japan, fewer medical institutions use social media than those in the United States and Western Europe. In addition, medical institutions using social media frequently used them as part of public relations activities. Some included messages that may violate medical advertising guidelines. To protect the reputation of medical institutions, it is considered necessary to formulate social media policies.

#### Social Media Accounts

In Japan, social media were rarely used by medical institutions, and it was considered that websites were mainly used for the dissemination of health information by medical institutions (Table 1). An online survey on health awareness among 3000 people showed that less than 5% used social networking sites as health information sources [41]. For this reason, even if a medical institution creates a social media account, only few users possibly refer to social media information from medical institutions. Because of a limited number of users, the number of “likes” and “followers” would not increase, and it would be difficult for medical institutions to ascertain the influence of using social media. As a result, medical institutions will interrupt the use of social media. In the United States, social media are an important source of information for using health information on the internet. According to a survey conducted in the United States in 2011, about one-fifth of approximately 23,000 respondents said that social media were the source of health information. In addition, one-third of respondents reported that social media are a reliable information source [42]. This viewpoint difference about social media between Japan and the United States may be reflected in the differences in social media utilization rates by medical institutions.

#### Social Media Utilization and Benefits

Social media have been used to maintain or improve peer-to-peer and clinician-to-patient communication, promote institutional branding, and improve the speed of interaction between and across different health care stakeholders in the health care field [43]. Patients may perceive that hospitals with social media activity are likely to offer advanced technologies and cutting-edge therapies [6].

In Japan, more than 50% of the social media comments sent by medical institutions were related to public relations activities. About a quarter of Facebook posts by hospitals were related to participation in academic conferences and the publication of academic papers (Figure 2). In text mining the Facebook accounts of hospitals, the frequency of “academic meeting” and “presentation” was high (Multimedia Appendix 4). Subgraphs related to conference presentations also appeared in the co-occurrence network (Figure 4). In particular, hospitals may have used social media to disseminate academic information. Additionally, an apology posted on Facebook by a hospital regarding the emergency discharge (quenching) of helium gas from a magnetic resonance imaging system was found (Multimedia Appendix 3). In this context, several reports have claimed that social media are a useful communication tool in emergency situations, such as disasters and accidents [44-51]. Further, social media may be useful when we want to share information urgently, because they have the advantage of immediacy compared to conventional media.

Social media have often been used for the purpose of health promotion and health education [5], and such health information may be used to improve public health as well. However, only a few medical knowledge and health information messages are disseminated by medical institutions in Japan. It may be even better to consider the season when health information is disseminated, as the number of monthly comments increased in December (Figure 1), a possible reason being the increased number of comments about Christmas as well as the year-end and New Year holidays. However, when medical institutions disseminate information on social media, it may be good to raise awareness not only about annual events but also about seasonal diseases. In fact, the spread of awareness on influenza vaccination using social media is common [52]. In addition, information on pollen allergy is provided using a mobile app [53].

Social media use by medical institutions involves mostly one-way communication, and few medical institutions respond to inquiries from the general public or patients via social media [8]. However, two-way communication with the general public and patients may meet patient needs that cannot be met through daily medical care and may thus help improve the provision of care [54,55].

#### Risks and Problems in Using Social Media

There are some problems with medical institutions using social media. These include concerns about patient privacy breaches, issues with the reliability and poor quality of information, and the obscuring of boundaries between health care professionals and patients [5,56].

When medical institutions disseminate information on social media, great care should be taken not to breach patient privacy as seemingly innocent comments can do so [57]. Even if the post does not contain a specific name, it may be possible to identify the patient by indirect information such as the name of the town where the patient lives, gender, or disease name [57]. Thus, medical institutions should be cautious when posting on social media, as these privacy breaches may occur unintentionally.

It is often difficult to tell who wrote health information on social media, which raises concerns regarding its accuracy and reliability [56]. Additionally, if medical institutions use social media, it will be necessary to clarify the boundaries between health care professionals and patients. Few doctors and medical institutions respond to “friend” requests from patients [56], but it is better to prescribe what to do when receiving “friend requests” in the social media policy in advance.

Moreover, when a medical institution uses social media, it may be necessary to create a social media policy not only to clarify the purpose of social media use but also to protect its reputation [56,58-60]. In this study, only 3 medical institutions disclosed their social media policies on their website. Thus, many medical institutions might not develop social media policies. In this context, damage to reputation and breach of patient privacy are matters of concern when medical institutions use social media [58]. Consequently, medical institutions should have clear objectives [59] and develop social media policies to avoid these risks.

#### Comparison With the Guidelines

In this survey, no content that violated patient privacy was extracted. However, some contents that could violate the guidelines were extracted. Of the hospital’s Facebook posts, 0.89% (12/1341) commented on being featured in the media. According to medical advertising guidelines, coverage announcements are also considered as advertising, and they are essentially restricted. Therefore, when sending information through social media, it would be necessary to refrain from commenting on whether their facility and staff are featured in newspapers, magazines, and other media. There was also a hospital Facebook account that sent company advertisements directly without disclosing conflicts of interest. This is considered ethically problematic. The Doctor’s Professional Ethics Guidelines stipulate that the relationship with medical providers should be appropriate [32]. In the website guidelines by the Japan Medical Association, “advertising by external sponsors” is listed as ineligible content [31]. Similarly, there are provisions regarding conflicts of interest in overseas guidelines; the British Medical Association social media usage guidelines require disclosure of conflicts of interest when doctors and medical students post information online [61].

Some clinics posted tweets emphasizing costs and matters not related to medical provision. An example is the toothbrush gift campaign when visiting the dental clinic, as well as discount campaigns such as medical checkups and whitening. In general, when a company uses a social medium for promotional purposes, coupons are often issued and discounts are announced on the social medium [62,63]. Therefore, if a medical institution uses social media like a company, it may be easy to disseminate messages on examinations and treatment fees and discounts. However, according to medical advertising guidelines, advertising that emphasizes costs is considered “Advertising that impairs dignity,” and such messages should not be disseminated. By disseminating such inappropriate messages, medical institutions not only receive a reprimand from health authorities but may also lose their good reputation.

The government should probably respond to messages on social media. In this study, referring to medical advertisement guidelines and the literature, we determined whether social media contents disseminated by medical institutions violated the guidelines. For some contents, it was difficult to determine whether they meet the guidelines. Governments might need to articulate the criteria for determining whether their contents are appropriate or inappropriate. The MHLW’s internet patrol and public notification regarding medical institutions’ websites are currently in execution [64,65]. In addition, it may be necessary to strengthen checks on inappropriate social media cases.

### Limitations

#### Sampling Methods

In this study, we randomly assigned a number to the list of medical institutions in Japan and extracted 300 small samples for each hospital and clinic. Compared to the actual number of medical institutions, these samples showed no statistically significant difference in the number of medical institutions by region, as presented in this study. In this study, regional bias may be possible, but it may be limited. However, the samples may not be representative of all Japanese medical institutions. These samples may be biased when examined in detail with prefectures and cities. Additionally, the characteristics and attributes of medical institutions may be biased. For a detailed study of social media usage in Japanese medical institutions in the future, it may be necessary to increase the sample size and reduce the confidence interval width. In addition, sampling methods such as stratified random sampling, cluster sampling, and multistage sampling should be used to obtain more representative samples [66].

#### Content Analysis

In this study, the classification of contents and the comparison with the medical advertising guidelines were made based on the consensus of 3 researchers. However, it did not preclude personal subjectivity; classification and comparison may be inconsistent, and objective evaluation will be necessary in future research. Further, measurement of intercoder reliability, which is fundamental and important in content analysis [67], is required for an objective evaluation. In addition, a thematic analysis approach such as topic modeling is required for objective categorization [68,69].

#### Factors Affecting Social Media Use in Medical Institutions

Regarding the use of social media by medical institutions, this study does not clarify the factors that led to the use of social media or the reasons why they were not used. Thus, the application of the unified theory of acceptance and use of technology and technology acceptance model may be necessary to examine the factors behind the use of social media in medical institutions [70].

#### Other Social Media

In this study, the target social media were limited to Facebook and Twitter. In future studies, it will be necessary to investigate the use of other platforms, such as blogs, wikis, LINE, and Instagram accounts of medical institutions. Blogs have been used since as early as 2004, and Wikipedia is often used in the medical community [43]. However, there are no reports of their usage at medical institutions in Japan, and the details remain unknown. LINE was developed in Japan [71], and its usage rate in Japan is high. According to a Ministry of Internal Affairs and Communications survey of 1500 people in 2016, Facebook usage was 32.3% and Twitter usage was 27.5%, whereas LINE usage was 67.0%, the highest [72]. In fact, it has been reported in a newspaper that a medical institution already uses LINE [73]. If medical institutions use LINE, messages pertaining to public relations and awareness activities may be more effectively distributed than via Facebook and Twitter. Instagram is a photo-sharing site that has been rapidly growing by the increasing number of users in recent years [74]. Medical institutions may be able to promote public relations activities by posting visually appealing images of them on Instagram. However, images that may violate medical advertising guidelines may be posted.

#### Lack of Cosmetic Surgery Clinics in the Sample

In this study, we investigated the actual use of social media by medical institutions throughout Japan but did not include cosmetic surgery clinics in the sample. Cosmetic surgery clinics might disseminate more advertisements than other specialties because many cosmetic surgeries are performed as part of free medical care, and the ratio of content may differ from this survey.

#### Necessity of a Longitudinal Study

The data presented in this study are cross-sectional at the time of the survey. Previous studies have shown that the use of social media by medical institutions has changed over time [7,75]. Therefore, in Japan, it will be necessary to observe social media usage by medical institutions over time.

### Conclusions

Social media usage by Japanese medical institutions is lower than that in the United States and Western European countries, and these media are mainly used for messages related to public relations. Some social media contents posted by medical institutions could conflict with medical advertising guidelines. In addition, few medical institutions have established social media policies. Due to deviations in usage rates from overseas and the characteristics of social media, it is necessary to consider social media other than Facebook and Twitter. This study may serve as a reference for medical institutions to guide social media usage and help improve medical website advertising in Japan.

## Appendix

Multimedia Appendix 1 Criteria for comparing social media content and guidelines, professional ethics.

Multimedia Appendix 2 Percentage of sample medical institutions by each region and percentage of actual medical institutions.

Multimedia Appendix 3 Examples of social media contents.

Multimedia Appendix 4 Number of frequencies of words in Facebook posts and tweets by hospitals and clinics top 50.

Multimedia Appendix 5 Examples of messages that may violate medical advertising guidelines and professional ethics.

## Abbreviations

MAU: Monthly Active Users
MHLW: Ministry of Health, Labour and Welfare
