# Supplementary material for: Use of Social Media by Hospitals and Clinics in Japan: Descriptive Study
Source: JMIR Med Inform. 2020 Nov 27;8(11):e18666. doi: 10.2196/18666 (PMC7732712; doi:10.2196/18666)
Supplement: Multimedia Appendix 1 [file medinform_v8i11e18666_app1.docx]

| **Multimedia Appendix 1 Criteria for comparing social media content and guidelines, professional ethics** | | |  |
| --- | --- | --- | --- |
| Evaluation items | Criteria | Applicable part of the guidelines | Example messages |
| Messages that cannot prove objective facts | that are not be realistic, such as “always” and “absolute” | False advertising | “We'll definitely succeed in any difficult case.” |
| Messages suggesting the superiority of the medical institution by comparison, exaggerated expressions of facility size, staffing, and/or medical provision | Expressions that may give a false sense of superiority compared to other facilities and markedly superior to other medical institutions  Comments that include superlative expressions such as “No. 1 in Japan,” “No. 1,” “Highest,” and other expressions that allude to superiority  Comments that may misrepresent the facility size, staffing, and medical services provided | Advertising that implies their superiority by comparison  Misleading advertising | “This hospital boasts the number of doctors in the prefecture.”  “Promising the best medical care!”  “This group operates nationwide. We provide the best medical care to public.” |
| Messages on safety | Messages highlighting treatment safety without providing objective data | Misleading advertising | “It's a relatively safe operation.” |
| Comparisons of photographs before and after operations | Pre-operative and post-operative photos that do not meet the limitation release requirement ^a^ | Photos before or after treatments that may mislead patients about the treatment methods and effects |  |
| Subjective or hearsay accounts | Patient stories, expert talks, etc. | Experiences of treatment or its effects based on the patient's subjectivity | Many people claim that “XX is effective.” |
| Invitation by matters not related to providing medical care | Messages for notice of presents in return for consultation | Advertising that impairs dignity | “We'll give XX to those who have received free consultation.”  “If you say ‘I saw the Tweet’ at the time of booking, we will present a XX!” |
| Emphasis on cost | Messages that emphasize campaigns, discounts, low prices | Advertising that impairs dignity | “Now running campaign”  “We offer XX therapy for 50% off for a limited time.” |
| Message that intentionally induces consultation | Message that induce consultation to a medical institution or implementation of a specific operation | Misleading advertising | “If you have these symptoms, you have a fatal condition. Visit a hospital right now.” |
| Professional qualification | Professional qualifications not permitted as medical advertisements | Not included in advertisable items |  |
| Medical department name | Departments not permitted as medical advertisements | Not included in advertisable items |  |
| Introduction in media | Messages about being featured in newspapers and magazines | By quoting or publishing articles in newspapers and magazines, discourses, theories, and experiences of doctors and scholars | “Doctor XX was introduced in the newspaper” |
| Satisfaction, therapeutic effect | Messages on customer satisfaction and treatment effectiveness | False advertising | “X% Satisfaction” (No basis or survey method is presented) |
| Regulations by other laws and other regulations | Messages that fall under other laws and regulations, such as the Pharmaceuticals and Medical Devices Act | Advertising prohibited by other laws or other advertising guidelines | “We can prescribe drug XX tablets (brand name).” |
| Messages against public order and morals | Messages that use obscene or cruel drawings or images or expressions that promote discrimination | Advertising that is contrary to public order or morality |  |
| Ethical issues | Ethically problematic messages regarding privacy violations and conflicts of interest disclosure | Refer to the “Doctors' Professional Ethics Guidelines” issued by the Japan Medical Association | “XX (celebrities, etc) visit our hospital!” |
|  |  |  |  |
| ^a^ For medical advertisements on the Internet except for banner advertisements and sponsor sites, listing contact information (E-mail address, Telephone number) eases some restrictions. If health insurance is not applied, additional information about treatment, costs, and risks should be required. | | | |
