# Supplementary material for: Use of Social Media by Hospitals and Clinics in Japan: Descriptive Study
Source: JMIR Med Inform. 2020 Nov 27;8(11):e18666. doi: 10.2196/18666 (PMC7732712; doi:10.2196/18666)
Supplement: Multimedia Appendix 2 [file medinform_v8i11e18666_app2.docx]

| **Multimedia Appendix 2 Percentage of sample medical institutions by each region and percentage of actual medical institutions** | | | | |  |
| --- | --- | --- | --- | --- | --- |
| Hospitals |  |  |  |  |  |
| Regional Bureau of Health and Welfare under the jurisdiction | Number of sample hospitals (X1) | Actual number of hospitals announced by the MHLW (Y1)^a^ | Percentage of samples (％)  (X1 / 300 * 100) | Percentage of actual hospitals (%)  (Y1 / 8412 * 100) | *P Value* |
| **Hokkaido** | 26 | 561 | 8.67 | 6.67 | .268 |
| **Tohoku** | 21 | 593 | 7.00 | 7.05 |  |
| **Kanto-Shinetsu** | 76 | 2347 | 25.33 | 27.90 |  |
| **Tokai-Hokuriku** | 34 | 903 | 11.33 | 10.73 |  |
| **Kinki** | 53 | 1327 | 17.67 | 15.78 |  |
| **Chugoku-Shikoku** | 31 | 645 | 10.33 | 7.67 |  |
| **Shikoku** | 12 | 468 | 4.00 | 5.56 |  |
| **Kyushu** | 47 | 1568 | 15.67 | 18.64 |  |
|  |  |  |  |  |  |
| Clinics |  |  |  |  |  |
| Regional Bureau of Health and Welfare under the jurisdiction | Number of sample clinics (X2) | Actual number of clinics announced by the MHLW (Y2)^b^ | Percentage of samples (％)  (X2 / 300 * 100) | Percentage of actual clinics (%)  (Y2 / 170080 * 100) | *P Value* |
| **Hokkaido** | 9 | 6318 | 3.00 | 3.71 | .958 |
| **Tohoku** | 18 | 10471 | 6.00 | 6.16 |  |
| **Kanto-Shinetsu** | 119 | 64951 | 39.67 | 38.19 |  |
| **Tokai-Hokuriku** | 37 | 21031 | 12.33 | 12.37 |  |
| **Kinki** | 50 | 31676 | 16.67 | 18.62 |  |
| **Chugoku-Shikoku** | 17 | 10430 | 5.67 | 6.13 |  |
| **Shikoku** | 11 | 5322 | 3.67 | 3.13 |  |
| **Kyushu** | 39 | 19881 | 13.00 | 11.69 |  |

^a, b^ Calculated based on reports released by the MHLW in 2017 [39]
