# Supplementary material for: Use of Social Media by Hospitals and Clinics in Japan: Descriptive Study
Source: JMIR Med Inform. 2020 Nov 27;8(11):e18666. doi: 10.2196/18666 (PMC7732712; doi:10.2196/18666)
Supplement: Multimedia Appendix 3 [file medinform_v8i11e18666_app3.docx]

| **Multimedia Appendix 3 Examples of social media contents ^a^** | | | |
| --- | --- | --- | --- |
| Hospitals or Clinics | Classification of comments | Social media | Messages |
| Hospitals | Health promotion | Facebook | Laparoscopic surgery for gastric and colorectal cancer has become widespread. This **** (hospital name) is also performing laparoscopic surgery with a 3D camera. More accurate lymph node dissection is being performed because neither the enlargement effect nor the perspective is lost. It is often emphasized that the wound is small, but the advantage of laparoscopic surgery is that it enables more precise operations in the stomach. |
|  |  | Twitter | According to epidemiological studies conducted in the United States, the lifetime prevalence of PTSD in adults is 4.0%, and the lifetime prevalence of complex PTSD is 3.3%. Complex PTSD is a concept that has emerged as a new international diagnostic standard in ICD-11.  (Omitted) |
|  | Participation in academic meetings, publications | Facebook | The **** conference held in **** is over. (Omitted) I am very grateful to the doctors for answering. |
|  |  | Facebook | (Omitted) When I submitted my paper to ****(journal name), I was immediately notified of Accept. I thought luck and timing were important. (Omitted) |
|  |  | Facebook | (Omitted) Dr ****'s report has been published to ****(journal name)! (Omitted) |
|  |  | Twitter | The 30th Annual Meeting of (conference name), the 2nd day. Please come to the symposium starting from **:**(time). (Omitted) |
|  | Public relations, news announcements | Facebook | (Omitted) We deeply apologize for the inconvenience and worry caused by the white smoke and explosion sound generated outside the MRI room. This is due to the emergency discharge of cooling gas from the MRI system, which does not damage the hospital building or equipment. (Omitted) |
|  |  | Facebook | Here is news of a medical open lecture! Do you have any symptoms such as crawling, swelling, swelling, or itching? Maybe it's a varicose vein. We will explain the current situation, diagnosis, and treatment of vascular surgical diseases from foot care to the feet, mainly for lower limb varicose veins and lower limb deep vein thrombosis! We look forward to your participation <(_ _)>  Lecturer: Vascular surgery Dr. **** Date: **** Place:****  Entry fee:**** Contact information:**** (Omitted) |
|  |  | Twitter | For a while since **** (date), we have been refraining from visiting inpatients. We will inform you again when visitation resumes. We ask for your understanding and cooperation in the context of preventing influenza infections among patients. (Omitted) |
|  |  | Twitter | The official website of ****(hospital name) has been updated. We have renewed our homepage to make it easier for everyone to use it and understand its content. (Omitted) |
|  | Recruitment | Facebook | News of recruitment of pharmacists For more information, see the recruitment information for https://****. |
|  |  | Facebook | Personnel changes dated *** ****(hospital name) Director Dr. **** (Surgery) (Omitted) |
|  |  | Twitter | [Recruitment] We are looking for general office staff. (Omitted) |
| Clinics | Health promotion | Facebook | (Omitted)  Do you use dental floss? ? There is dirt that cannot be removed by brushing with a toothbrush hard! ! The hard-to-remove dirt leads to “cavities” and “periodontal disease”! !  (Omitted) |
|  |  | Twitter | Recently, “high-quality HbA1c” has been required.  HbA1c reflects blood glucose levels for the past month or two. It is a representative index used worldwide, but lower is not better.  HbA1c reduction using oral hypoglycemic drugs (oral medications) or insulin injections has a hypoglycemic risk. |
|  | Participation in academic meetings, publications | Facebook | Today, I have been attending the **** lifelong training in ****. |
|  | Public relations, news announcements | Facebook | About year-end and New Year holidays We will be closed as follows without permission. (Omitted) |
|  |  | Twitter | Closed on the morning of ****(date) due to snow. In the afternoon, regular medical treatment is scheduled. |
|  | Recruitment | Facebook | Staff recruitment Why don't you work with a new and beautiful eye clinic that has just opened in ****(month)? (Omitted) |
| ^a^ Japanese Facebook posts and tweets were translated into English. | | | |
