# Supplementary material for: Use of Social Media by Hospitals and Clinics in Japan: Descriptive Study
Source: JMIR Med Inform. 2020 Nov 27;8(11):e18666. doi: 10.2196/18666 (PMC7732712; doi:10.2196/18666)
Supplement: Multimedia Appendix 4 [file medinform_v8i11e18666_app4.docx]

| **Multimedia Appendix 4 Number of frequencies of words in Facebook posts and tweets by hospitals and clinics top 50 ^a^** | | | | | | | | | |  |  |  |  |  |  |  |
| --- | --- | --- | --- | --- | --- | --- | --- | --- | --- | --- | --- | --- | --- | --- | --- | --- |
| Hospital |  |  |  |  |  |  |  |  | Clinic |  |  |  |  |  |  |  |
| Facebook |  |  |  |  | Twitter |  |  |  | Facebook |  |  |  |  | Twitter |  |  |
| Order | Japanese | English | Frequency |  | Japanese | English | Frequency |  | Order | Japanese | English | Frequency |  | Japanese | English | Frequency |
| 1 | 先生 | doctor | 2504 |  | 更新 | update | 582 |  | 1 | 先生 | doctor | 238 |  | 診療 | consultation | 169 |
| 2 | 月 | month | 1940 |  | ブログ | blog | 527 |  | 2 | 月 | month | 216 |  | 月 | Monday | 134 |
| 3 | 病院 | hospital | 1703 |  | [A病院] | [hospital A] | 522 |  | 3 | 休診 | closed | 158 |  | 水 | Wednesday | 84 |
| 4 | 参加 | participation | 1702 |  | RT | RT | 517 |  | 4 | 院長 | director | 148 |  | 休診 | closed | 73 |
| 5 | 医療 | medical | 1361 |  | 月 | month | 384 |  | 5 | 診療 | consultation | 118 |  | 矯正 | orthodontic | 53 |
| 6 | 開催 | hold | 1327 |  | おはよう | good morning | 366 |  | 6 | [名字 A] | [name A] | 109 |  | 更新 | update | 48 |
| 7 | 行う | do | 1264 |  | [B病院] | [hospital B] | 288 |  | 7 | [名字 B] | [name B] | 94 |  | 予約 | reservation | 48 |
| 8 | 研修 | training | 1170 |  | 病院 | hospital | 259 |  | 8 | お願い | please | 82 |  | ブログ | blog | 45 |
| 9 | 年 | year | 990 |  | [****会] | [medical corporation name] | 235 |  | 9 | 行う | do | 81 |  | 本日 | today | 44 |
| 10 | 思う | think | 837 |  | 今日 | today | 222 |  | 10 | 当院 | our clinic | 67 |  | お願い | please | 37 |
| 11 | 学会 | academic meeting | 815 |  | タイトル | title | 210 |  | 11 | 患者 | patient | 65 |  | 通常 | normal | 30 |
| 12 | 地域 | regional | 802 |  | 研修 | training | 198 |  | 12 | 医師 | doctor | 63 |  | お知らせ | information | 28 |
| 13 | 患者 | patient | 762 |  | 看護 | nursing | 177 |  | 13 | 今日 | today | 62 |  | 相談 | counseling | 27 |
| 14 | 当院 | our hospital | 753 |  | お楽しみ会 | fun party | 170 |  | 14 | [名字 C] | [name C] | 60 |  | 金曜日 | Friday | 25 |
| 15 | 発表 | presentation | 746 |  | 年 | year | 167 |  | 15 | 必要 | necessary | 59 |  | 電話 | call | 24 |
| 16 | 日本 | Japan | 730 |  | 開催 | hold | 155 |  | 16 | オメガ | omega | 58 |  | 皆様 | everyone | 22 |
| 17 | 研究 | research | 635 |  | 演奏 | musical performance | 143 |  | 17 | 予約 | reservation | 57 |  | 時間 | time | 22 |
| 18 | 治療 | therapy | 620 |  | (月) | Monday | 133 |  | 18 | 火 | Tuesday | 56 |  | お待ち | wait | 21 |
| 19 | 講演 | lecture | 619 |  | 医療 | medical | 132 |  | 19 | 木 | Thursday | 52 |  | 治療 | therapy | 21 |
| 20 | 診療 | consultation | 600 |  | 参加 | participation | 132 |  | 20 | 治療 | therapy | 51 |  | 気軽 | freely | 20 |
| 21 | 医師 | doctor | 597 |  | 天気 | weather | 124 |  | 21 | スタッフ | staff | 50 |  | 研修 | training | 20 |
| 22 | 今回 | this time | 528 |  | 雨 | rain | 113 |  | 22 | 効果 | effect | 50 |  | 午後 | afternoon | 20 |
| 23 | 平成 | Heisei | 518 |  | 当院 | our hospital | 113 |  | 23 | 菌 | bacteria | 49 |  | （月） | Monday | 18 |
| 24 | 皆様 | everyone | 494 |  | 外来 | outpatient | 111 |  | 24 | 摂取 | intake | 49 |  | 午前 | morning | 18 |
| 25 | センター | center | 492 |  | 子ども | child | 109 |  | 25 | 本日 | today | 49 |  | 無料 | free | 18 |
| 26 | 活動 | activity | 486 |  | リハビリ | rehabilitation | 103 |  | 26 | 時間 | time | 48 |  | 迷惑 | inconvenience | 18 |
| 27 | 教授 | professor | 469 |  | 朝 | morning | 103 |  | 27 | 療法 | therapy | 47 |  | 医院 | clinic | 16 |
| 28 | お願い | please | 461 |  | 花 | flower | 101 |  | 28 | 多い | high | 46 |  | 血糖 | blood sugar | 16 |
| 29 | 今後 | from now on | 451 |  | 平成 | Heisei | 97 |  | 29 | 食事 | diet | 45 |  | 勝手 | without permission | 16 |
| 30 | 救急 | emergency | 419 |  | 職員 | staff | 97 |  | 30 | 栄養素 | nutrient | 44 |  | 糖尿 | glucosuria | 16 |
| 31 | 医局 | medical office | 375 |  | ADHD | ADHD | 94 |  | 31 | 栄養 | nutrition | 43 |  | 変更 | change | 16 |
| 32 | 時間 | time | 362 |  | 行う | do | 94 |  | 32 | 環境 | environment | 43 |  | おかけ | annoying | 15 |
| 33 | 今年 | this year | 360 |  | 続く | continue | 92 |  | 33 | 思う | think | 43 |  | 患者 | patient | 15 |
| 34 | ケア | care | 358 |  | ご覧 | see | 91 |  | 34 | クリニック | clinic | 42 |  | 臨時 | temporary | 15 |
| 35 | 予定 | planning | 352 |  | 皆様 | everyone | 90 |  | 35 | 腸 | intestine | 42 |  | 行う | do | 14 |
| 36 | お知らせ | information | 349 |  | 庭園 | garden | 90 |  | 36 | 土 | Saturday | 41 |  | 誠に | really | 14 |
| 37 | ママ | mother | 348 |  | 病棟 | ward | 85 |  | 37 | 症状 | symptom | 39 |  | 理解 | understanding | 14 |
| 38 | スタッフ | staff | 337 |  | 咲く | bloom | 85 |  | 38 | 水 | Wednesday | 39 |  | 可能 | possible | 13 |
| 39 | 考える | consider | 333 |  | 桜 | cherry-blossom | 84 |  | 39 | お知らせ | information | 38 |  | 歯科 | dental | 13 |
| 40 | 看護 | nursing | 329 |  | 実習 | practice | 84 |  | 40 | 人 | people | 37 |  | 都合 | circumstance | 13 |
| 41 | 臨床 | clinical | 324 |  | 先生 | doctor | 83 |  | 41 | 通常 | normal | 36 |  | 土 | Saturday | 13 |
| 42 | 外科 | surgery | 317 |  | 明日 | tomorrow | 81 |  | 42 | 歯科 | dental | 35 |  | 当院 | our clinic | 13 |
| 43 | 眼科 | ophthalmology | 316 |  | 注意 | careful | 81 |  | 43 | 金 | Friday | 33 |  | 明日 | tomorrow | 12 |
| 44 | 土 | Saturday | 315 |  | 予定 | planning | 80 |  | 44 | 出る | appear | 33 |  | 考える | consider | 11 |
| 45 | 皆さん | everyone | 313 |  | 研究 | research | 80 |  | 45 | [クリニック A] | [clinic A] | 32 |  | ニュース | news | 10 |
| 46 | 介護 | nursing care | 312 |  | 今週 | this week | 79 |  | 46 | 良い | good | 32 |  | 掃除 | cleaning | 10 |
| 47 | 看護師 | nurse | 310 |  | 降る | [It rains or snows] | 78 |  | 47 | 脂肪酸 | fatty acid | 30 |  | 木 | Thursday | 10 |
| 48 | 勉強 | study | 310 |  | 治療 | therapy | 77 |  | 48 | 出来る | can | 30 |  | 予定 | planning | 10 |
| 49 | 学生 | student | 307 |  | 雪 | snow | 74 |  | 49 | 皮膚 | skin | 30 |  | 休む | closure | 9 |
| 50 | 多く | many | 305 |  | 気温 | temperature | 74 |  | 50 | ドクター | doctor | 29 |  | 参加 | participation | 9 |
| ^a^ Japanese words were translated into English. | | | | | | | | |  |  |  |  |  |  |  |  |
