# Supplementary material for: Use of Social Media by Hospitals and Clinics in Japan: Descriptive Study
Source: JMIR Med Inform. 2020 Nov 27;8(11):e18666. doi: 10.2196/18666 (PMC7732712; doi:10.2196/18666)
Supplement: Multimedia Appendix 5 [file medinform_v8i11e18666_app5.docx]

| **Multimedia Appendix 5 Examples of messages that may violate medical advertising guidelines and professional ethics ^a^** | | | | |
| --- | --- | --- | --- | --- |
| Hospitals or Clinics | Social media | Messages | Applicable evaluation items  (Refer to Multimedia Appendix 1) | Applicable part of the guidelines |
| Hospitals | Facebook | On **** (date), an article about **** surgery was published in the serial article "****" in the **** Shimbun. (Omitted) Please read it. | Introduction in media | By quoting or publishing articles in newspapers and magazines, discourses, theories, and experiences of doctors and scholars |
|  | Facebook | Featured in the opening interview of the ****(month) issue of Monthly **** (journal title). | Introduction in media | (Same as above) |
|  | Facebook | (Omitted) We will share the introduction of the sponsoring company here. **** (product name), it is stable | Regulations by other laws and regulations  Ethical issues | Advertising prohibited by other laws or other advertising guidelines  Refer to the “Doctors' Professional Ethics Guidelines” issued by the Japan Medical Association |
|  | Twitter | Published on ****(publisher name)'s “****(journal title)” **** special page. https: // **** | Introduction in media | By quoting or publishing articles in newspapers and magazines, discourses, theories, and experiences of doctors and scholars |
|  | Twitter | (Limited-time discount) We offer a 10% discount on the overnight dock (usually **** yen) at **** yen (tax included) from **** to ***** (month) only. (Omitted) | Emphasis on cost | Advertising that impairs dignity |
| Clinics | Facebook | (Omitted) As the teeth whiten, the sense of cleanliness goes up!  In our clinic we want everyone to experience "Low price" and "safety" We offer whitening! (Omitted) | Messages on safety  Emphasis on cost  Messages suggesting the superiority of the medical institution by comparison, exaggerated expressions of facility size, staffing, and/or medical provision | Misleading advertising  Advertising that impairs dignity  Advertising that implies their superiority by comparison |
|  | Twitter | (Omitted) If you are using Twitter, please let us know because we are giving a toothbrush set as a gift (Omitted) | Invitation by matters not related to providing medical care | Advertising that impairs dignity |
|  | Twitter | Xylitol cupcakes will be presented to those who have been consulted between **** and **** (date)! (Omitted) | Invitation by matters not related to providing medical care | (Same as above) |
|  | Twitter | (Omitted) We have a campaign for cleaning and whitening teeth. Please take this opportunity. (Omitted) | Emphasis on cost | (Same as above) |
| ^a^ Japanese Facebook posts and tweets were translated into English. | | |  |  |
